# Cardiorespiratory Responses and Prediction of Peak Oxygen Uptake during the Shuttle Walking Test in Healthy Sedentary Adult Men

**DOI:** 10.1371/journal.pone.0117563

**Published:** 2015-02-06

**Authors:** Camila D. C. Neves, Ana Cristina Rodrigues Lacerda, Vanessa K. S. Lage, Liliana P. Lima, Sueli F. Fonseca, Núbia C. P. de Avelar, Mauro M. Teixeira, Vanessa A. Mendonça

**Affiliations:** 1 Universidade Federal dos Vales do Jequitinhonha e Mucuri, Programa Multicêntrico de Pós- Graduação em Ciências Fisiológicas, Sociedade Brasileira de Fisiologia, Diamantina, Minas Gerais, Brazil; 2 Universidade Federal dos Vales do Jequitinhonha e Mucuri, Laboratório de Inflamação e Metabolismo, Diamantina, Minas Gerais, Brazil; 3 Universidade Federal de Santa Catarina, Departamento de Fisioterapia, Araranguá, Santa Catarina, Brazil; 4 Universidade Federal de Minas Gerais, Laboratório de Imunofarmacologia, Belo Horizonte, Minas Gerais, Brazil; Clinica Universidad de Navarra, SPAIN

## Abstract

**Background:**

The application of the Shuttle Walking Test (SWT) to assess cardiorespiratory fitness and the intensity of this test in healthy participants has rarely been studied. This study aimed to assess and correlate the cardiorespiratory responses of the SWT with the cardiopulmonary exercise testing (CEPT) and to develop a regression equation for the prediction of peak oxygen uptake (VO2 peak) in healthy sedentary adult men.

**Methods:**

In the first stage of this study, 12 participants underwent the SWT and the CEPT on a treadmill. In the second stage, 53 participants underwent the SWT twice. In both phases, the VO2 peak, respiratory exchange ratio (R), and heart rate (HR) were evaluated.

**Results:**

Similar results in VO2 peak (P>0.05), R peak (P>0.05) and predicted maximum HR (P>0.05) were obtained between the SWT and CEPT. Both tests showed strong and significant correlations of VO2 peak (r = 0.704, P = 0.01) and R peak (r = 0.737, P<0.01), as well as the agreement of these measurements by Bland-Altman analysis. Body mass index and gait speed were the variables that explained 40.6% (R2 = 0.406, P = 0.001) of the variance in VO2 peak. The results obtained by the equation were compared with the values obtained by the gas analyzer and no significant difference between them (P>0.05) was found.

**Conclusions:**

The SWT produced maximal cardiorespiratory responses comparable to the CEPT, and the developed equation showed viability for the prediction of VO2 peak in healthy sedentary men.

## Introduction

Assessment of functional capacity or cardiorespiratory fitness (CRF) is commonly performed with the goal of providing parameters for the prescription and preparation of exercise programs, as well as providing information about reduced exercise tolerance under various pathological conditions [1,2]. The assessment of CRF through maximal tests on treadmills or cycle ergometers (cardiopulmonary exercise testing—CEPT), with direct measurement of maximal oxygen uptake (VO_2max_ or VO_2_ peak) is considered the gold-standard measurement; however, this measurement is not always possible, because it has high costs and requires a specialized laboratory and trained personnel [3]. Thus, the development and use of field tests and equations to predict VO_2_ peak allow for simple, secure, affordable and indirect assessment of CRF.

Among field tests, we emphasize the Shuttle Walking Test (SWT), which is a test of incremental structure and symptom-limited, with the pace dictated externally [4]. The incremental format of the SWT causes a progressive increase in the workload, and with this increase, a strong correlation with the performance of CEPT and SWT has been observed in cardiopulmonary patients [5–7].

In recent years, the application of the SWT to assess the CRF of healthy individuals has been implemented [8–10]; however, the application and intensity of this test in this population is still relatively unknown and studied [11–13]. When evaluating the intensity of the SWT by means of the maximum heart rate (HR_max_) achieved at the end of the test, the results of these studies have shown differences in maximal and submaximal rates. Compared with the VO_2_ peak values achieved during the SWT, the predicted values of this measurement in CEPT appear to be similar, but such responses must be confirmed with the completion of CEPT since this comparison was performed only with the VO_2_ peak values estimated for the population studied [13]. Furthermore, all these studies were conducted with elderly or middle-aged participants, and because increasing age directly influences CRF, this data cannot be extrapolated to younger participants. Thus, the present study aimed to evaluate and correlate the cardiorespiratory responses during the SWT and a CEPT, in order to classify the intensity of SWT, and to develop a regression equation to predict VO_2_ peak in healthy sedentary young and middle-aged men. We hypothesized that the SWT would promote maximal cardiorespiratory responses comparable to the CEPT, and the reference equation would be viable for the prediction of VO_2_ peak in healthy sedentary men.

## Methods

This study involved healthy male participants aged 18–45 years old that were recruited from the local community. To be included in the study, the participants had to meet the following criteria: self-report of no present acute or chronic diseases; be eutrophic according to the body mass index (BMI between 18.5 and 24.9 kg/m^2^); and to currently not be using corticosteroid medications. Moreover, since the SWT was developed for cardiopulmonary patients, i.e., with low CRF, only sedentary subjects (who did not perform physical activity for 30 minutes or more at least three times per week [3]) were included. To meet the objectives, this study was divided into two stages. The first stage aimed to evaluate the intensity of the SWT and the second stage aimed to develop a regression equation for the prediction of VO_2_ peak. This study followed the declaration of Helsinki. The Ethics and Research Committee of the Federal University of the Jequitinhonha and Mucuri, Brazil, approved this study (protocol 108/12). All participants gave written, informed consent.

### First stage procedures

In the first stage, 12 volunteers went to the laboratory on three consecutive days at the same time period each day. On the first day, the body composition was assessed by weight, height, BMI and body fat percentage measures, and familiarization was performed. The weight and height were measured on an anthropometric mechanical scale, the BMI was calculated as the weight divided by height squared, and the body fat percentage was estimated by measuring of chest, abdominal and thigh skinfold thickness using a plicometer [14,15]. Familiarization consisted of testing that would be performed on consecutive days to reduce the effect of learning on later visits. On the second and third days, the SWT and CEPT were applied. The testing order was randomized and balanced. The entire procedure took place during a single day shift and the subjects were given specific instructions. The subjects were to avoid physical activity and any intake of caffeine and alcohol in the 24 hours prior to testing, to get at least 8 hours of sleep the night before, to eat a light meal and to ingest 500 ml of water in the two hours before the tests [3]. On the days of testing, subjects were asked about their compliance with the recommendations above and about possible complications or changes in their daily routines.

As described by Singh et al. [4], to perform the SWT, the participants were instructed to walk a distance of 10 meters around a marking between two cones, placed 0.5 m from each endpoint. The walking speed at which the participant should walk was dictated by a sound played from a CD that was originally generated by a microcomputer. Each minute, the walking speed increased by 0.17 m/s, with an initial speed of 0.5 m/s. The test was finished when the volunteer was not able to maintain the required speed (more than 0.5 m from the cone), at the request of the volunteer, or for some other reported symptom (dyspnea, dizziness, vertigo, angina). The original protocol consisted of 12 levels (1020 m); however, as suggested by the literature, we used a protocol of 15 levels (1500 m) to evaluate healthy participants, in order to prevent the ceiling effect [10,11]. Before and after the test, heart rate (HR, measured by a heart rate monitor), blood pressure (measured by a mercury sphygmomanometer cuff and a stethoscope) and rating of perceived exertion (RPE, Borg scale, range 6–20) were measured [16]. Additionally, during the testing, the laps were recorded to calculate the distance and gait speed reached at the last full level.

The CEPT was performed on a treadmill using a protocol based on the progression of the SWT. This protocol consisted of 1-minute stages, with speed increasing every minute without increasing the incline of the treadmill. The initial velocity was 0.5 m/s, and it increased by 0.17 m/s at each stage. Before, during, and after the test, heart rate, blood pressure and RPE were measured as described above. The criteria for stopping the test was as follows: systolic blood pressure (SBP) greater than 210 mm Hg; diastolic blood pressure greater than 120 mm Hg; sustained decrease in SBP; angina; dyspnea; cyanosis; nausea; dizziness; or by the request of the volunteer [3].

### Second stage procedures

In the second stage, 53 volunteers went to the laboratory at two different days. On the first day, the body composition measurements were obtained as described in the first stage. On the second day, the participants went to the laboratory for two SWTs, with an interval of 30 minutes between them. Completion of two SWTs with this interval had been suggested to reduce the effects of the learning test [11,17]. For the data analysis, the results of the test in which the volunteer obtained the greatest distance covered were used. As with the first stage, the entire procedure took place during a single day shift: the subjects were instructed to follow all the recommendations for the practice of physical tests, and prior to completion of the tests, the subjects were asked about their compliance with the recommendations and about possible complications or changes in their daily routines.

### Physiological responses

During the tests of the two stages of this study, the exhaled gases were collected using a gas analyzer via the portable telemetry system (K4b2, Cosmed, Rome, Italy). Among other variables, oxygen uptake (VO_2_), respiratory quotient (R) and HR breath-by-breath were monitored. The data was filtered and was defined as VO_2_ peak and R peak with the highest value obtained from the arithmetic mean of the log intervals of 30s and maximum HR (HR_max_) as the highest HR value recorded during the test [18,19]. Predicted HR_max_ was calculated by the equation HR_max_ = 220 – age [20].

### Validation of the reference equation

To validate the equation, a different group of healthy males, composed of 20 individuals, was selected according to the same inclusion criteria of the study. This group completed the SWT as described in the preceding stages. Likewise, the VO_2_ peak obtained by the gold-standard method (gas analyzer) was compared with the VO_2_ peak predicted by the reference equation.

### Statistical analysis

The statistical analysis was performed using the statistical packages SPSS 20.0 (Inc., USA) and GraphPad Prism 4 (Inc., USA). In the first stage, the normality of data was checked by the Shapiro-Wilk test and the differences among measured variables were determined by paired-t-test for variables with normal distribution or the Wilcoxon test for variables with non-normal distribution. Pearson’s coefficient of correlation was performed to study the correlation between variables and the agreement between tests was assessed by Bland-Altman analysis. The sample size was calculated based on the study by Singh et al. [7] and was identified by the needs of 10 participants. In the second stage, the normality of data was checked by the Kolmogorov-Smirnov test and for compiling the reference equation, the linear multiple regression analysis was performed to identify the predictors of the dependent variable. The sample size was estimated based on the relationship between the number of variables to be included in the multiple regression analysis and the minimum number of observations required, indicating at least 52 participants in order to develop a linear model containing up to 4 variables. At the end of the regression analysis, the paired t-test was utilized to compare the means of the results obtained by the reference equation with the measured values ​​of VO_2_ peak obtained using the gas analyzer. Moreover, the validation of the reference equation was evaluated in an additional group of 20 volunteers: the values of VO2 peak obtained by the reference equation were compared with the measured values ​​of VO_2_ peak obtained by the gas analyzer using the paired t-test. The level of statistical significance was *P*<0.05.

## Results

### First stage

**Fig 1:**
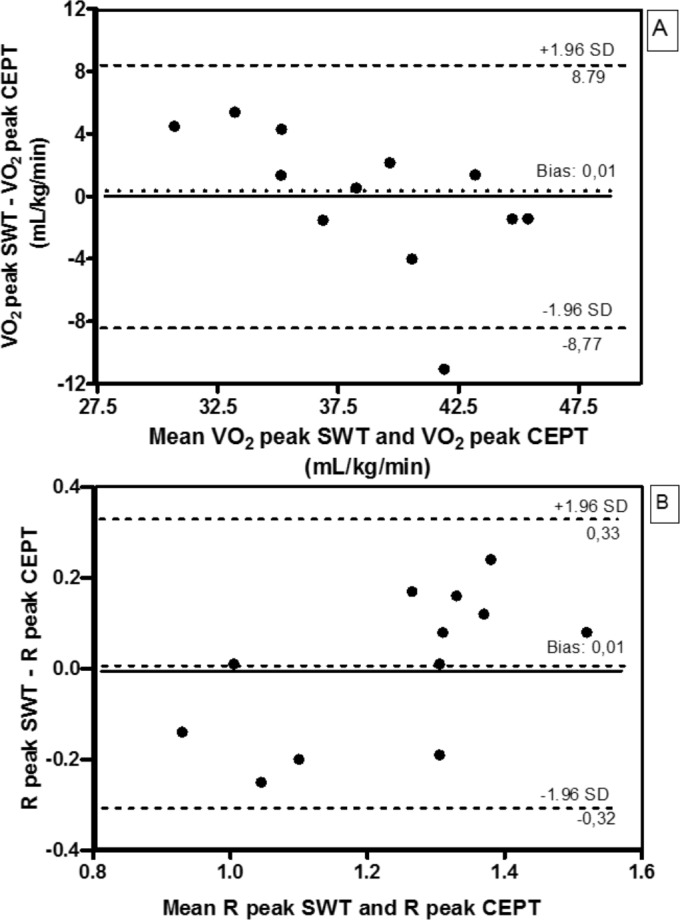
Agreement between VO2 peak and R peak obtained in the SWT and CEPT. Bland-Altman plot of the difference between the VO_2_ peak of the SWT and CEPT plotted against the mean VO_2_ peak of the SWT and CEPT (A) and difference R peak of the SWT and CEPT plotted against the mean R peak of the SWT and CEPT (B). SWT = Shuttle Walking Test; CEPT = cardiopulmonary exercise testing; VO_2_ = oxygen uptake; R = respiratory exchange ratio.

Twelve participants, with a mean age 25.1 ± 5.8 years old, weight of 71.5 ± 5.6 kg, height of 1.77 ± 0.06 m, BMI of 22.9 ± 1.9 kg/m^2^ and body fat percentage of 12.0 ± 5.5%, participated in the first stage of the study. The RPE and cardiorespiratory responses obtained at the end of the SWT and CEPT are presented in Table 1. Similar results in VO_2_ peak, R peak, and predicted HR_max_ were found. The RPE at the end of the test was significantly higher in the CEPT. Strong and significant correlations in VO_2_ peak (*r* = 0.704, *P* = 0.01) and R peak (*r* = 0.737, *P*<0.01) were found between the tests. The Bland-Altman analysis also showed agreement between the results for VO_2_ peak and R peak on the SWT and CEPT (Fig. 1A and B).

**Table 1:** Comparison between the results of cardiorespiratory variables and RPE at the end of the test, obtained in SWT and CEPT.

	SWT	CEPT	*P*–value
VO_2_ peak (mL/kg/min)	38.7 ± 3.8	38.7 ± 6.3	0.993^†^
R peak	1.3 ± 0.24	1.3 ± 0.14	0.877^†^
HR_max_ (% predicted)	97.6 ± 6.1	98.3 ± 4.1	0.700^†^
RPE (points)	17.7 ± 1.6	19.7 ± 0.5	0.007*^¥^

The data is presented as mean (SD). *P<0.05. SWT = Shuttle Walking Test; CEPT = cardiopulmonary exercise testing; VO_2_ = oxygen uptake; R = respiratory exchange ratio; HR = heart rate; RPE = rating of perceived exertion. ^†^Paired-t test; ^¥^Wilcoxon test.

### Second stage

The characteristics of the 53 participants that participated in the second stage of the study are shown in Table 2. Age, BMI, distance walked and gait speed were the demographic, anthropometric and physical performance variables selected for the preparation of the reference equation, respectively. The univariate analysis showed that the VO_2_ peak correlated significantly with distance walked (*r* = 0.405, *P*<0.01), gait speed (*r* = 0.446, *P* = 0.000) and BMI (*r* = -0.515, *P* = 0.000). There was no significant correlation with age (*r* = -0.175, *P* = 1.08). A model of stepwise linear multiple regressions showed that BMI and gait speed explained 40.6% (*R* ^*2*^ = 0.406, *P* = 0.001) of the variance in VO_2_ peak. There was no interaction of the VO_2_ peak with distance walked and age. The 95% Confidence Interval for unstandardized coefficients were the constants (15.149–55.514), BMI (-1.606 to -0.528) and gait speed (4.746–16.950). The reference equation for the VO_2_ peak in the SWT was:

$${VO}_{2}peak(predicted)=37.44-\left(1.081\times BMI\right)+\left(10.151\times gaitspeed\right)$$

**Table 2:** General characteristics of the participants of the second stage.

General characteristics	*n* = 53
Age (years)	32.42 ± 7.38
Weight (kg)	69.3 ± 8.2
Height (m)	1.75 ± 0.06
BMI (kg/m^2^)	22.66 ± 2.1
Body fat percentage (%)	11.5 ± 5.6
Distance walked (m)	1069 ± 152
Gait Speed (m/s)	2.36 ± 0.2
HR_max_ (% predicted)	98.75 ± 6.1

The data is presented as mean (SD). BMI = body mass index. HR = heart rate.

The results obtained by the equation of VO_2_ peak with the values ​​obtained by the gas analyzer, showed no significant difference between them (VO_2_ peak [predicted] = 36.66 ± 3.22 mL/kg/min; VO_2_ peak [gas analyzer] = 36.72 ± 5.1 mL/kg/min, *P*>0.05).

### Validation of the reference equation

The characteristics of the other group composed of 20 healthy sedentary adult men were: age 23.7 ± 3.5 years old, weight of 69.9 ± 9.2 kg, height of 1.76 ± 0.07 m, BMI of 22.7 ± 2.1 kg/m^2^ and body fat percentage of 10.5 ± 4.9%. When the reference equation was applied in this group, the VO_2_ peak obtained using the gold-standard method was not statistically different from the VO_2_ peak predicted by the elaborated equation (39.39 mL/kg/min and 39.33 mL/kg/min, *P* = 0.95; respectively).

## Discussion

This was the first study to compare the cardiorespiratory responses between the SWT with CEPT in healthy sedentary participants. During the SWT, the participants reached the values of the HR_max_ and of the R peak, thus classifying the SWT as a high-intensity test (maximum) since the values of the HR_max_ ≥ 90% and of the R peak ≥ 1:10 indicate a maximal test [18,19]. Despite being significantly lower, the RPE also reached values characteristic of the maximum tests [18]. Strong and significant correlations between VO_2_ peak and R peak and the agreement of these variables were obtained by both tests. Similar results have been observed with the values of the VO_2_ peak on the SWT and with the values of the VO_2_ peak on the CEPT performed by patients with COPD [5,7], idiopathic pulmonary fibrosis [21] and heart failure [6,22]. These results support the proposal that the SWT is a field test of maximal effort that provides objective measurement of CRF on cardiopulmonary patients.

Due to its recent application in healthy participants, the determination of intensity of the SWT remains controversial in this population. Dourado and Guerra [12] and Jurgensen et al. [11] applied the SWT to healthy participants of both sexes, with mean ages of 57 and 58 years old respectively, and they reported values of predicted HR_max_ at the end of the test, which might indicate the SWT to be a submaximal test. Different results of other studies in which participants of both sexes, with similar ages, have also been observed and the studies showed values of predicted HR_max_ ranging from 94% [13] to 99% [17], thus indicating that the SWT could be featured as the maximum test. Such differences could have been due to variances in the samples studied, since the studies included active, sedentary, and obese participants and patients with stable heart disease. The application of the SWT in young adults has been reported in only two studies; however, aspects of these works should be noted. In the study by Probst et al. [17], although it applied criteria for inclusion of participants 18–83 years old, the mean age of the participants represented mostly a sample of middle-aged adults. Moreover, in that study, the participants were characterized as apparently healthy; yet, a lot of the participants in their sample had some comorbidity, such as hypertension, arthritis, diabetes among others. These factors could have influenced the test´s results. In the study by Seixas et al. [23], young adult participants, reached mean values submaximal of predicted HR_max_, however, the sample consisted of physically active participants, recruited from a gym, who performed the SWT at 12 levels. Because the participants were young, healthy, and physically active, the use of the SWT at the 12 levels might have generated insufficient overloading for this sample; thus, the results might have been underestimated. From the data on the walking distance in the present study, we confirmed the necessity of using the protocol with 15 levels for our sample, which consisted of healthy sedentary young and middle-aged adults, since the average distance walked exceeded the maximum distance of the 12 levels. To date, no studies have compared the cardiorespiratory responses of the SWT with CEPT in healthy participants. Given the information above, and based on the data of the present study, we suggest that the SWT provides valid measurement and assessment of CRF in healthy sedentary participants, and as in cardiopulmonary patients, the SWT in young and middle-aged people could be considered a field test of maximal effort.

The reference equation developed in this study explained 40.6% of the variance in VO_2_ peak. Gait speed as a major determinant of VO_2_ peak was also observed in recent studies that developed reference equations for the prediction of VO_2_ peak during the application of the SWT in healthy, middle-aged, and elderly participants [8,13]. Amongst athletes and young adults attending fitness classes, the variance in predicted VO_2_ peak in the Shuttle Run Test, a precursor test of the SWT, was also determined by gait speed [24]. As with gait speed, BMI influenced the prediction of VO_2_ peak. The correlation between the two measurements was negative, which can be explained by an inverse linear relationship between body composition and functional capacity. The influence of body composition on VO_2_ peak and the distance walked on the SWT has been reported in the literature [8,11,17]. Age and distance walked did not affect VO_2_ peak. As the gait speed recorded was equivalent to that of the last completed level, this finding showed a strong correlation with the distance walked and thus had a greater influence on VO_2_ peak in stepwise linear multiple regression analysis. Similar to our findings, age did not influence VO_2_ peak in young adults who underwent the Shuttle Run Test [24]. Despite CRF being directly influenced by age, this influence appears only to be present in the population above the age of 40 years old, mainly due to reductions in lean body mass and increased body fat [25].

Although the equation developed in this study could be explained by moderate variance, the VO_2_ peak values obtained by the equation and the values of VO_2_ peak obtained by the gas analyzer were similar, thus demonstrating the feasibility of applying the equation to healthy, sedentary young and middle-aged men. It is noteworthy that reference equations for distance walked during the SWT in healthy participants with variances of this magnitude have been published in the literature [11]. Lastly, a limitation of this study is that our sample consisted only of male participants and since the influence of sex on VO_2_ peak could not be evaluated, the reference equation elaborated has applicability only in men.

## Conclusions

It is concluded that in healthy, sedentary young and middle-aged men, the SWT produced results similar to those of the CEPT with regards to cardiorespiratory responses, and the reference equation demonstrated viability for the prediction of VO_2_ peak in this population.
